# Meteorological Report for October, 1879

**Published:** 1879-11

**Authors:** H. Hall

**Affiliations:** Corp’l. Signal Service U.S. A.; Atlanta, Ga.


### METEOROLOGICAL REPORT FOR OCTOBER, 1879.

| RAIN. DATE | RAIN. IN. | Mean daily Barometer. | Mean daily Thermometer | Mean daily Humidity. | Maximum Thermometer | Minimum Thermometer | Prevailing direction of Wind. | Maximum hourly velocity of Wind, (Daily.) |
|---|---|---|---|---|---|---|---|---|
| 1 | 0 | 30.232 | 73.0 | 77.7 | 79 | 66 | E. | ......... |
| 2 | 0 | 30 176 | 74.5 | 60.0 | 83 | 66 | E. | ......... |
| 3 | 0 | 30.123 | 73.7 | 60.3 | 82 | 63 | E. | ......... |
| 4 | 0 | 30 053 | 75.2 | 55.6 | 82 | 68 | E. | ......... |
| 5 | 05 | 30.052 | 71.0 | 78.7 | 75 | 66 | E. | ......... |
| 6 | 03 | 30.001 | 70 0 | 71 0 | 76 | 66 | E. | ......... |
| 7 | 66 | 30.028 | 70 2 | 91.7 | 73 | 69 | S. E. | ......... |
| 8 | 02 | 30.131 | 73.5 | 84.7 | 80 | 69 | S. E. | ......... |
| 9 | 0 | 30.122 | 74.0 | 82.0 | 80 | 69 | S. E. | ......... |
| 10 | ...... | 30 041 | 73.2 | 77.3 | 83 | 69 | S. E. | ......... |
| 11 | 14 | 30.069 | 69.5 | 63 5 | 73 | 66 | E. | ......... |
| 12 | 21 | 30.133 | 67.5 | 82.3 | 73 | 63 | E. | ......... |
| 13 | 01 | 30.109 | 70.2 | 76.3 | 78 | 62 | E. | ......... |
| 14 | 0 | 30 103 | 69.5 | 76 0 | 76 | 63 | E. | ......... |
| 15 | 0 | 30 023 | 70.5 | 72 7 | 76 | 63 | E. | ......... |
| 16 | 1.08 | 29 900 | 70.0 | 95.0 | 72 | 67 | E. | ......... |
| 17 | 49 | 29.952 | 73.0 | 90 0 | 76 | 70 | S. E. | ......... |
| 18 | 1.48 | 29.976 | 65.0 | 89.3 | 73 | 61 | S. W. | ......... |
| 19 | 0 | 30.015 | 59.0 | 77.7 | 65 | 54 | N. W. | ......... |
| 20 | 43 | 30 159 | 51.0 | 84.0 | 59 | 50 | E. | ......... |
| 21 | 07 | 30.103 | 53.7 | 94.3 | 56 | 50 | E. | ......... |
| 22 | 70 | 30.089 | 60.0 | 93.7 | 63 | 64 | E. | ......... |
| 23 | 0 | 30.180 | 57.0 | 68.7 | 67 | 49 | N. W. | ......... |
| 24 | 0 | 30.367 | 46.5 | 44.3 | 57 | 40 | N. W. | ......... |
| 25 | 0 | 30.568 | 44.0 | 43 0 | 51 | 38 | E. | ......... |
| 26 | 0 | 30.515 | 48 0 | 50.0 | 56 | 36 | N. E. | ......... |
| 27 | 06 | 30 154 | 51.0 | 85.0 | 53 | 47 | N. E. | ......... |
| 28 | 01 | 29.907 | 57.7 | 54 3 | 67 | 53 | N. W. | ......... |
| 29 | 0 | 29 974 | 59.7 | 36.7 | 69 | 45 | N. W. | ......... |
| 30 | 0 | 29.957 | 61.0 | 52.3 | 71 | 52 | W. | ......... |
| 31 | 0 | 30.187 | 53.2 | 44.0 | 63 | 45 | N. W. | ......... |
| Monthly Means. | | 30 109 | 64.0 | 71.3 | 70.6 | 58.0 | | |

Highest Barometer, 30.640 on the 26th.
Lowest Barometer, 29.819, on the 28th.
Monthly range of Barometer, 0.821.
Highest Temp. 83°, on 2nd and 10th. Lowest Temp. 36°, on 26th.
Monthly range of Temperature, 47°.
Greatest daily range of Temperature, 24°, on the 29th.
Lowest daily range of Temperature, 4°, on the 7th.
Mean of Maximum Temperatures, 70 6.
Mean of Minimum Temperatures, 58.0.
Mean daily range of Temperature, 12.6. Total rainfall, 5.44 inches.
Prevailing wind, East. Total movement of wind, 75.61 miles.
Maximum velocity of wind and direction, 33 miles per hour, East, 16th.
Number Cloudy days on which rain fell, 11.
Number of cloudy days on which no rain fell, 6.
Total number of days on which rain fell, 16.

H. HALL, Corp'l. Signal Service U.S. A.

Atlanta, Ga., Oct. 1, 1879.

